# Author Correction: Mutualist and pathogen traits interact to affect plant community structure in a spatially explicit model

**DOI:** 10.1038/s41467-020-16612-y

**Published:** 2020-05-26

**Authors:** John W. Schroeder, Andrew Dobson, Scott A. Mangan, Daniel F. Petticord, Edward Allen Herre

**Affiliations:** 1 Smithsonian Tropical Research Institute, Balboa Ancon, Republic of Panama; 2 Department of Ecology, Evolution, and Marine Biology, University of California, Santa Barbara, Santa Barbara, CA USA; 3 Princeton University, Princeton, NJ USA; 4 Santa Fe Institute, Hyde Park Road, Santa Fe, NM USA; 5 Washington University, St. Louis, MO USA

**Keywords:** Microbial ecology, Theoretical ecology, Biodiversity, Forest ecology

Correction to: *Nature Communications* 10.1038/s41467-020-16047-5, published online 5 May 2020.

The original version of this Article omitted the following from the Acknowledgements:

Support for this work was provided by Simons Foundation award 429440 (WTW).

This has now been corrected in both the PDF and HTML versions of the Article.

