# Supplementary material for: Mutualist and pathogen traits interact to affect plant community structure in a spatially explicit model
Source: Nat Commun. 2020 May 5;11:2204. doi: 10.1038/s41467-020-16047-5 (PMC7200732; doi:10.1038/s41467-020-16047-5)
Supplement: Supplementary file 1 — Supplementary Information [file 41467_2020_16047_MOESM1_ESM.pdf]

Supplementary Information

Mutualist and pathogen traits interact to affect plant community structure in a spatially explicit model

Schroeder et al.

Supplementary Note 1. Model philosophy

In building our simulation, we decided to include many parameters, rather than distilling the simulation to the fewest parameters possible. Using a broad search through parameter space, and an ensemble learning model (Random Forest), we identify the several parameters that are the most important determinants of specific outcomes (e.g. diversity maintenance or negative plant-soil feedback). We employed this approach in order to systematically consider many possible explanations for how microbial community dynamics can create observed patterns of plant diversity, and plant-soil feedback. The benefit of this approach is that it reduces a priori bias toward any specific explanation for any given phenomenon. The downside of this approach is that it is more computationally intensive.

An additional strength of the approach described above is that it will easily accommodate more information about the natural history and dynamics of plant-microbiome interactions. In the present manuscript, even though we explore the influence of 17 possible parameters on plant-soil feedback dynamics, we make many simplifying assumptions (described in the Methods). As we learn more about the direct, and indirect, interactions between pathogens, mutualists, and their environment, we can incorporate additional information into our modelling framework. Using the ensemble learning approach, we can then determine whether added complexity substantively changes the dynamics of the simulation.

Supplementary Table 1. Expanded parameter descriptions

Parameter	Definition
s_f	Host specificity of mutualists (f = m) and pathogens (f = p). When s_m = 1, mutualists are complete generalists, and a microbe has an equal affinity for all hosts. When s_m = 0.3, the affinity of mutualists for non-preferred hosts is 30% of the affinity of mutualists for preferred hosts. Host affinities of mutualists[1,2] and pathogens[3,4] have been independently investigated in multiple studies. Such studies have shown that mutualists and pathogens can often associate with many host species, but usually have host-specific effects.
γ_f	Fecundity of microbes, represented as a proportion of microbes in any given cell that are dispersed as propagules.
b_f	Exponent of power law distribution. This indicates the dispersal limitation of both microbial guilds (f = m, f = p), or plants (f = t).<br>Dispersal kernels have been estimated for trees[5] and fungal spores[6]. Power law functions have been shown to fit both plant and fungal dispersal[5,6].
g	The impact of microbes on host survival. Higher values of g correspond to simulations in which a given change in the abundance of pathogens or mutualists has a greater effect on seedling recruitment.
h	Relative contribution of the mutualist community to seedling recruitment probability.
ζ	Relative fitness of least fit plant species. The fitness values of plant species (i.e. ζ_j where j is the plant species), are evenly distributed between ζ and 1.
c_f	Exponent that scales the competitive ability of a microbe according to its effect on host survival. As c_f increases, the competitive effect of a microbe decreases when it is associating with a non-preferred host. We assume that c_f is greater than zero, meaning that the competitive ability of a microbe associating with a given host decreases as its effect on host survival decreases.
q_f	Exponent that scales intrinsic growth rate of microbes with host affinity. When the value is negative, microbial populations rapidly crash after their preferred host dies. High values of this parameter allow for a microbe to persist at a site after its preferred host dies. We interpret high values of q_p as a representation of taxon's ability to escape competition by

	occupying alternative life history strategies (e.g. plant pathogens that can persist as saprotrophs or endophytes when they cannot exploit a living host).
r_f	Intrinsic growth rate of mutualists and pathogens.
α_f	Competition coefficients of mutualists and pathogens

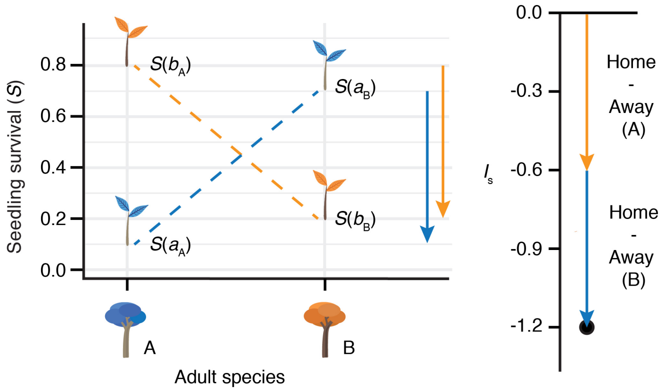

Supplementary Figure 1. Components of the plant-soil feedback interaction coefficient. The plant-soil feedback interaction coefficient can be calculated for two plant species, A and B, to determine whether the effect of plants on their local soil environment contributes to plant coexistence[7,8]. The metric is calculated as follows:

$$I_s = S(a_A) + S(b_B) - \big(S(a_B) + S(b_A)\big)$$

where $S(a_A)$ and $S(b_B)$ are the respective survival probabilities of seedlings of species A and B grown beneath conspecific adults (home performance), and $S(a_B)$ and $S(b_B)$ are the survival probabilities of seedlings of species A and B grown beneath adults of the other species (away performance). Each of these values can be measured empirically to calculate I_s using potted, or field experiments[9]. Many studies measure PSF using a home-away approach, $S(a_A) - S(a_H)$, where $S(a_H)$ is the average performance of species A beneath heterospecifics[10]. Although this approach captures one component of PSF, it cannot predict whether any two species will coexist, because it does not account for the effect of species A on heterospecifics. In the figure above**,** seedling survival probabilities yield a negative interaction coefficient (i.e. negative PSF). Negative feedback occurs when plants alter their local soil microbiomes to have a relatively more detrimental effect on their own seedlings than the seedlings of other species[11]. Empirical studies show that a local accumulation of relatively species-specific pathogens beneath adult trees creates negative feedback[10,12,13].

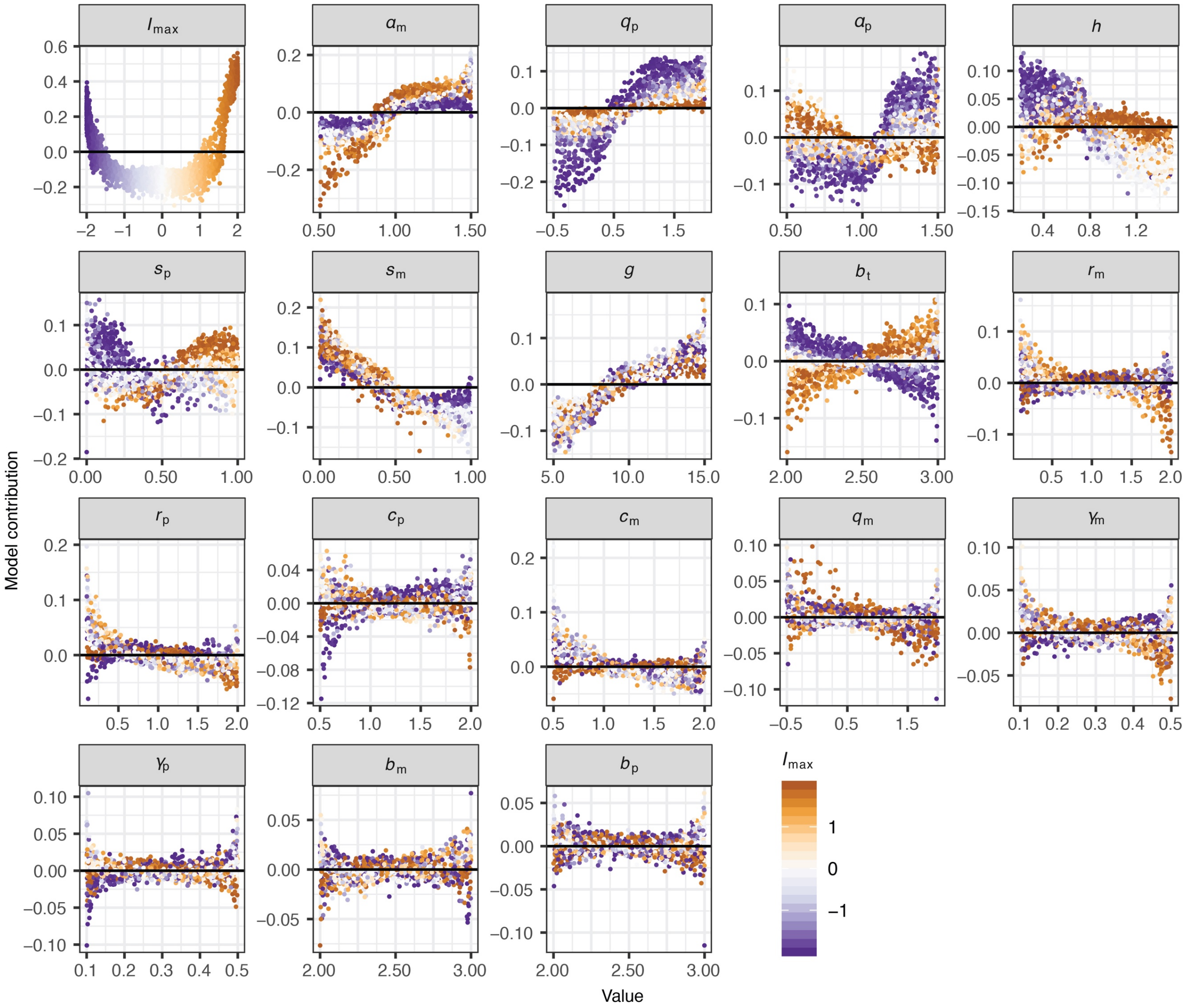

Supplementary Figure 2. Conditional feature contributions - predictors of coexistence. Point colour represents the proportion of decision trees that predict that all plant species will coexist for the duration of the simulation (i.e. no extinctions).

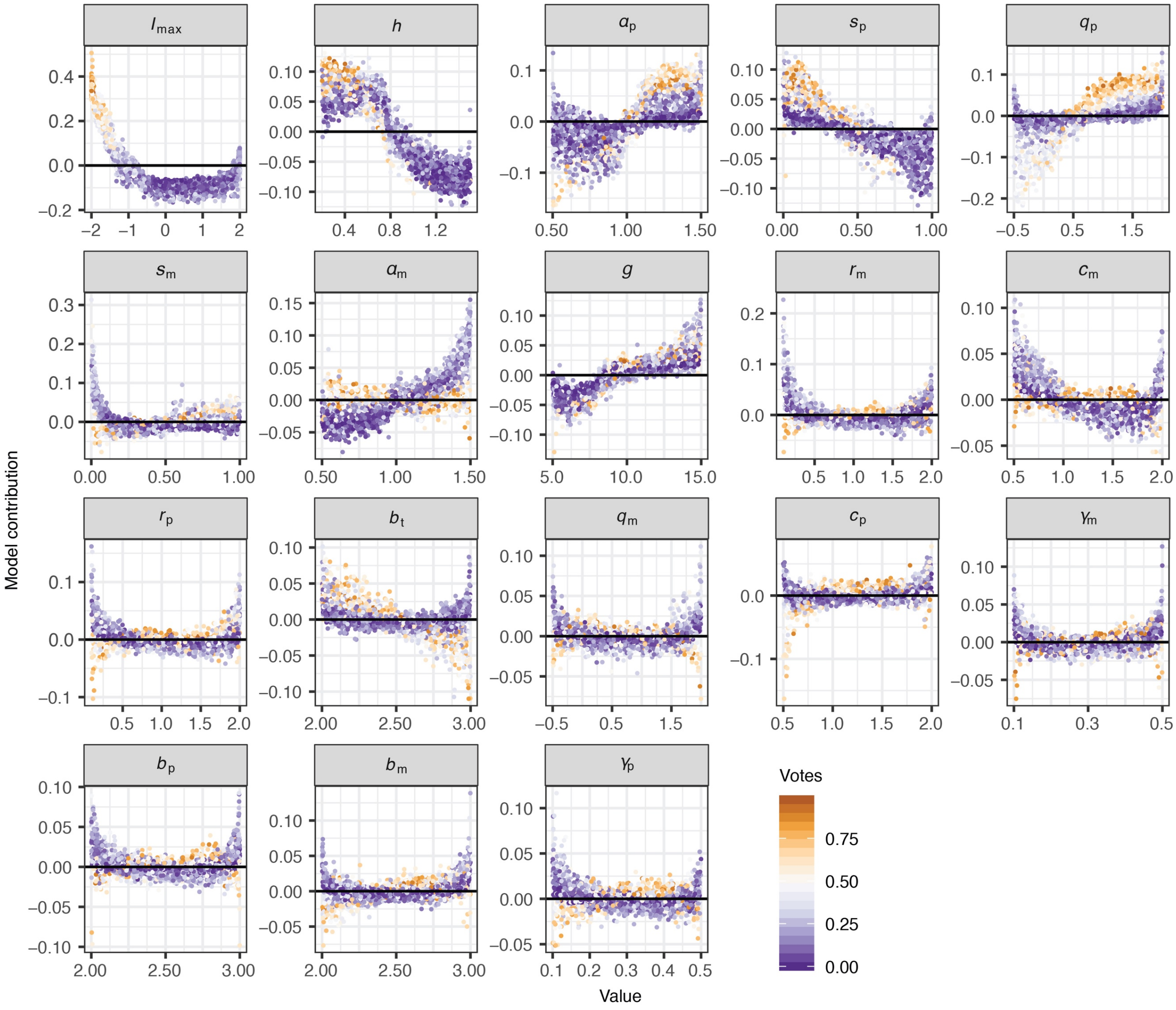

Supplementary Figure 3. Conditional feature contributions – equilibrium. Specifically, conditional feature contributions from a random forest model using all predictors to determine whether the abundance of the fittest plant species will reach a stable equilibrium ($P_e > 0.1$) and coexistence (i.e. 5 species coexist until the end of the simulation). P_e represents the equilibrium metric generated from a two-sided t-test, with n = 60 time intervals. No correction for multiple testing was performed, because the P value was intended as a metric. Point colour represents the proportion of decision trees predicting whether all species coexist under equilibrium at the end of the simulation (i.e. the equilibrium metric $P_e > 0.1$).

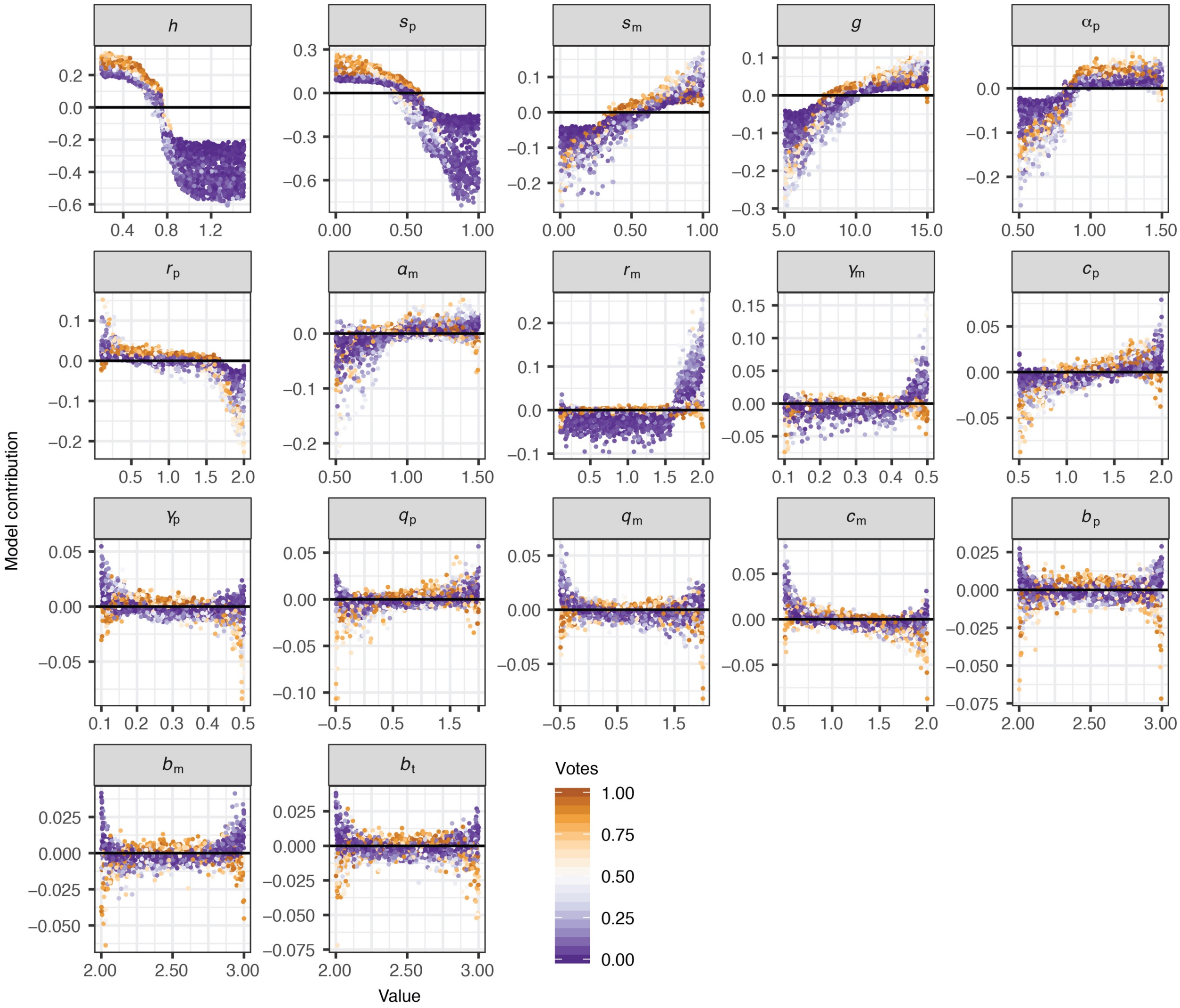

Supplementary Figure 4. Conditional feature contributions – negative feedback. Specifically, conditional feature contributions of all predictors of whether strong feedback will develop (random forest 2). Point colour represents the proportion of decision trees that predict that I_{max} will be negative.

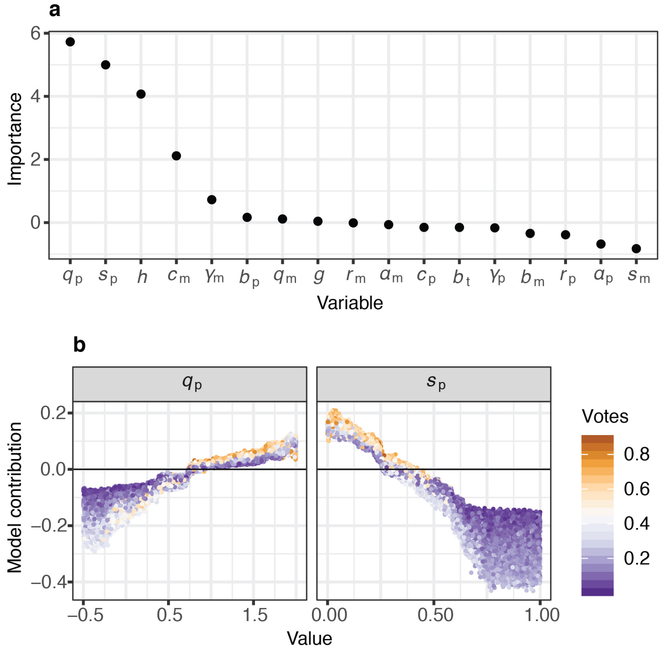

Supplementary Figure 5. Important predictors – PSF-abundance correlation. Specifically, results from a random forest classifier using each variable to predict whether a strong positive correlation ($\rho > 0.8$) will develop between host abundance and feedback. **a**, Importance of each variable (mean decrease in accuracy). **b**, Conditional feature contribution of variables predicting the relationship between host abundance and feedback strength. In addition to the relative effect of mutualists on seedling survival, h, and the relative host affinity of mutualists and pathogens, s_f, the most important variable in determining whether a strong positive correlation between host abundance and feedback developed was q_p (the exponent scaling pathogens' intrinsic growth rate with their effect on a non-target host). Effectively, this variable decreases the rate at which pathogens decay in abundance at a site after their preferred host is replaced by a non-target host. As q_p increases, pathogens are more able to maintain higher abundances after their host dies. See Supplementary Figure 4 for the conditional feature contributions of every variable.

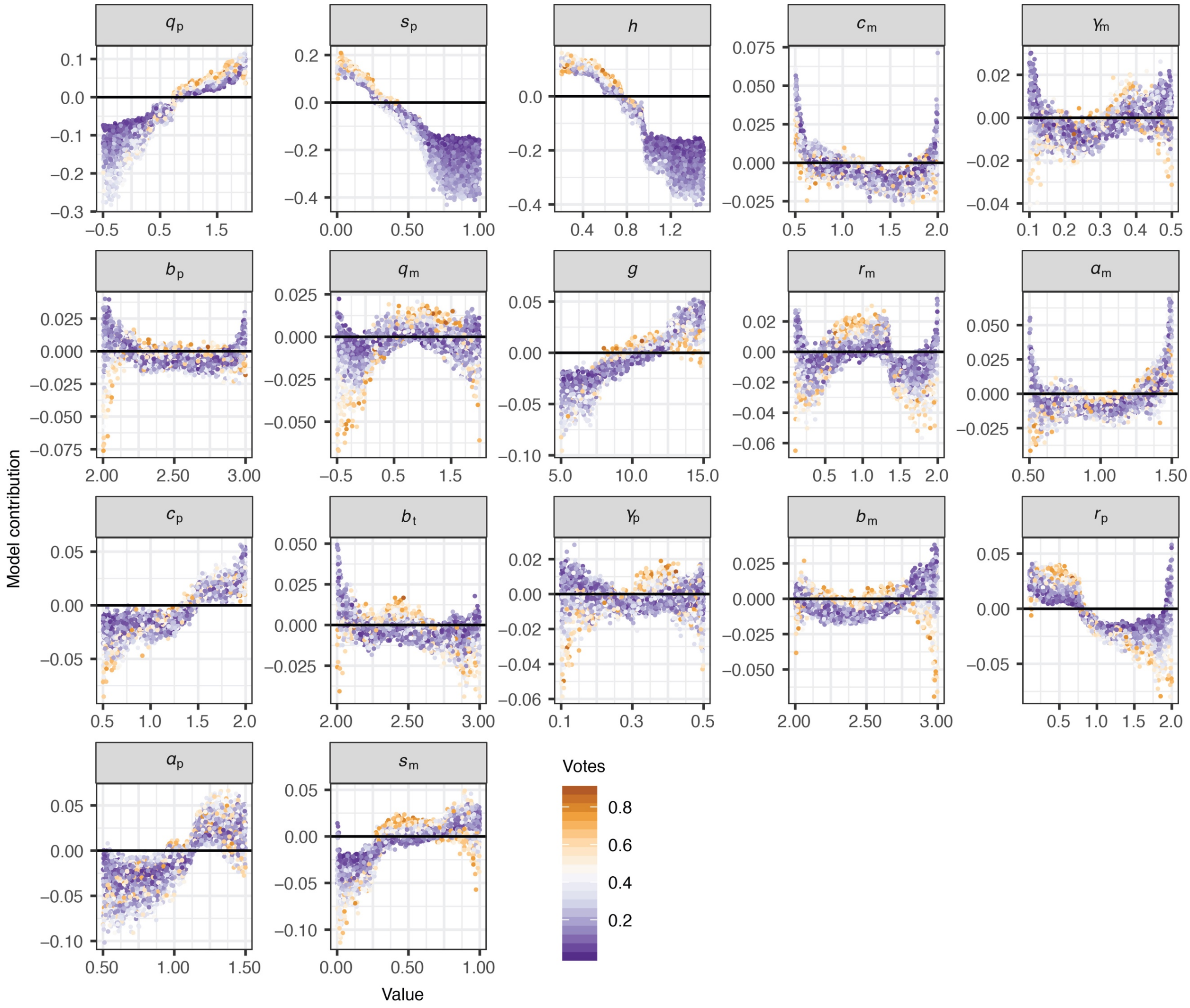

Supplementary Figure 6. Conditional feature contributions – PSF-abundance correlation. Specifically, conditional feature contributions of all predictors of whether a positive correlation between host abundance and PSF will develop. Point colour represents the proportion of decision trees predicting a strong positive correlation between host abundance and feedback.

Supplementary References

1. Mangan, S. A., Herre, E. A. & Bever, J. D. Specificity between Neotropical tree seedlings and their fungal mutualists leads to plant-soil feedback. *Ecology* **91**, 2594–2603 (2010).
2. Kiers, E. T., Lovelock, C. E., Krueger, E. L. & Herre, E. A. Differential effects of tropical arbuscular mycorrhizal fungal inocula on root colonization and tree seedling growth: implications for tropical forest diversity. *Ecol. Lett.* **3**, 106–113 (2000).
3. Sarmiento, C. *et al.* Soilborne fungi have host affinity and host-specific effects on seed germination and survival in a lowland tropical forest. *Proc. Natl. Acad. Sci.* **114**, 11458–11463 (2017).
4. Gilbert, G. S. & Webb, C. O. Phylogenetic signal in plant pathogen-host range. *Proc. Natl. Acad. Sci.* **104**, 4979–4983 (2007).
5. Bullock, J. M. *et al.* A synthesis of empirical plant dispersal kernels. *J. Ecol.* **105**, 6–19 (2017).
6. Farber, D. H., De Leenheer, P. & Mundt, C. C. Dispersal kernels may be scalable: Implications from a plant pathogen. *J. Biogeogr.* **46**, 2042–2055 (2019).
7. Bever, J. D., Westover, K. M. & Antonovics, J. Incorporating the soil community into plant population dynamics: the utility of the feedback approach. *J. Ecol.* **85**, 561–573 (1997).
8. Crawford, K. M. *et al.* When and where plant-soil feedback may promote plant coexistence: a meta-analysis. *Ecol. Lett.* **22**, 1274–1284 (2019).
9. Mangan, S. A. *et al.* Negative plant-soil feedback predicts tree-species relative abundance in a tropical forest. *Nature* **466**, 752–755 (2010).
10. Klironomos, J. N. Feedback with soil biota contributes to plant rarity and invasiveness in communities. *Nature* **417**, 67–70 (2002).

11. Bever, J. D., Mangan, S. A. & Alexander, H. M. Maintenance of plant species diversity by pathogens. *Annu. Rev. Ecol. Evol. Syst.* **46**, 305–325 (2015).

12. Bever, J. D., Platt, T. G. & Morton, E. R. Microbial population and community dynamics on plant roots and their feedbacks on plant communities. *Annu. Rev. Microbiol.* **66**, 265–283 (2012).

13. Packer, A. & Clay, K. Soil pathogens and spatial patterns of seedling mortality in a temperate tree. *Nature* **404**, 278–281 (2000).
