# Supplementary material for: Mutualist and pathogen traits interact to affect plant community structure in a spatially explicit model
Source: Nat Commun. 2020 May 5;11:2204. doi: 10.1038/s41467-020-16047-5 (PMC7200732; doi:10.1038/s41467-020-16047-5)
Supplement: Supplementary file 3 — Reporting Summary [file 41467_2020_16047_MOESM3_ESM.pdf]

natureresearch

Corresponding author(s): John W Schroeder

Last updated by author(s): Apr 6, 2020

Reporting Summary

Nature Research wishes to improve the reproducibility of the work that we publish. This form provides structure for consistency and transparency in reporting. For further information on Nature Research policies, see Authors & Referees and the Editorial Policy Checklist .

Statistics

For all statistical analyses, confirm that the following items are present in the figure legend, table legend, main text, or Methods section.

n/a	Confirmed	
☐	☒	The exact sample size (n) for each experimental group/condition, given as a discrete number and unit of measurement
☒	☐	A statement on whether measurements were taken from distinct samples or whether the same sample was measured repeatedly
☐	☒	The statistical test(s) used AND whether they are one- or two-sided<br>*Only common tests should be described solely by name; describe more complex techniques in the Methods section.*
☐	☒	A description of all covariates tested
☐	☒	A description of any assumptions or corrections, such as tests of normality and adjustment for multiple comparisons
☐	☒	A full description of the statistical parameters including central tendency (e.g. means) or other basic estimates (e.g. regression coefficient) AND variation (e.g. standard deviation) or associated estimates of uncertainty (e.g. confidence intervals)
☒	☐	For null hypothesis testing, the test statistic (e.g. F, t, r) with confidence intervals, effect sizes, degrees of freedom and P value noted<br>*Give P values as exact values whenever suitable.*
☒	☐	For Bayesian analysis, information on the choice of priors and Markov chain Monte Carlo settings
☒	☐	For hierarchical and complex designs, identification of the appropriate level for tests and full reporting of outcomes
☐	☒	Estimates of effect sizes (e.g. Cohen's d, Pearson's r), indicating how they were calculated

Our web collection on statistics for biologists contains articles on many of the points above.

Software and code

Policy information about availability of computer code

Data collection	All data were generated from a custom simulation run on R version 3.6.3 The code can be accessed on GitHub at https://github.com/johnwschroeder/PlantMicrobeSimulation, or at http://doi.org/10.5281/zenodo.3742143.
Data analysis	All data were analyzed using R version 3.6.3. The code can be accessed on GitHub at https://github.com/johnwschroeder/PlantMicrobeSimulation, or at http://doi.org/10.5281/zenodo.3742143

For manuscripts utilizing custom algorithms or software that are central to the research but not yet described in published literature, software must be made available to editors/reviewers. We strongly encourage code deposition in a community repository (e.g. GitHub). See the Nature Research guidelines for submitting code & software for further information.

Data

Policy information about availability of data

All manuscripts must include a data availability statement. This statement should provide the following information, where applicable:

- Accession codes, unique identifiers, or web links for publicly available datasets
- A list of figures that have associated raw data
- A description of any restrictions on data availability

Simulation results that support the findings of this study are available on GitHub at https://github.com/johnwschroeder/PlantMicrobeSimulation, or at http://doi.org/10.5281/zenodo.3742143.

Field-specific reporting

Please select the one below that is the best fit for your research. If you are not sure, read the appropriate sections before making your selection.

Ecological, evolutionary & environmental sciences study design

All studies must disclose on these points even when the disclosure is negative.

Study description	Here, we describe results from a spatially explicit theoretical simulation of plants interacting with microbial mutualists and pathogens (i.e. microbial plant-soil feedback). Specifically, we ask which combinations of microbial trait values (1) create a frequency dependent rare species advantage that can maintain overall plant diversity, and (2) generate the commonly observed positive correlation between host abundance and PSF (i.e. common plants exhibit weaker negative PSF than rare plants).
Research sample	We generated all data using custom simulation code available at https://github.com/johnwschroeder/PlantMicrobeSimulation. Where applicable, we performed statistical analyses in which each simulation run was an independent sample.
Sampling strategy	For random forest analyses that used simulation parameter values to predict model outcomes, we used results from 16K simulation runs with random parameter values to conduct our analyses. We deemed this was a sufficient number of simulations, because independent batches of runs produce identical conclusions. For results that focus on a specific parameterization, we conducted repeated independent optimization procedures that identified similar parameterizations.
Data collection	We generated all of our data using the simulation described in the manuscript.
Timing and spatial scale	NA
Data exclusions	NA
Reproducibility	We provide all code necessary to independently repeat our simulations. We also provide the simulation results used to conduct the analyses described in the manuscript (archived at http://doi.org/10.5281/zenodo.3742143).
Randomization	NA
Blinding	NA

Did the study involve field work? ☐ Yes ☒ No

Reporting for specific materials, systems and methods

We require information from authors about some types of materials, experimental systems and methods used in many studies. Here, indicate whether each material, system or method listed is relevant to your study. If you are not sure if a list item applies to your research, read the appropriate section before selecting a response.

Materials & experimental systems

n/a	Involved in the study	
☒	☐	Antibodies
☒	☐	Eukaryotic cell lines
☒	☐	Palaeontology
☒	☐	Animals and other organisms
☒	☐	Human research participants
☒	☐	Clinical data

Methods

n/a	Involved in the study	
☒	☐	ChIP-seq
☒	☐	Flow cytometry
☒	☐	MRI-based neuroimaging
